# Porcine epidemic diarrhea virus strain FJzz1 infection induces type I/III IFNs production through RLRs and TLRs-mediated signaling

**DOI:** 10.3389/fimmu.2022.984448

**Published:** 2022-07-25

**Authors:** Pengfei Chen, Junrui Zhu, Jiarong Yu, Ruilin Liu, Mengqin Lao, Lingxue Yu, Fei Gao, Yifeng Jiang, Changlong Liu, Wu Tong, Huili Liu, Guangzhi Tong, Yanjun Zhou

**Affiliations:** ^1^ Department of Swine Infectious Diseases, Shanghai Veterinary Research Institute, Chinese Academy of Agricultural Sciences, Shanghai, China; ^2^ Institute of Animal Husbandry and Veterinary, Shanghai Academy of Agricultural Science, Shanghai, China; ^3^ Jiangsu Co-innovation Center for Prevention and Control of Important Animal Infectious Diseases and Zoonoses, Yangzhou University, Yangzhou, China

**Keywords:** porcine epidemic diarrhea virus, ISG, JAK-STAT pathway, IFN-I/III, innate immunity

## Abstract

Interferons (IFNs) including type I/III IFNs are the major components of the host innate immune response against porcine epidemic diarrhea virus (PEDV) infection, and several viral proteins have been identified to antagonize type I/III IFNs productions through diverse strategies. However, the modulation of PEDV infection upon the activation of the host’s innate immune response has not been fully characterized. In this study, we observed that various IFN-stimulated genes (ISGs) were upregulated significantly in a time- and dose-dependent manner in LLC-PK1 cells infected with the PEDV G2 strain FJzz1. The transcriptions of IRF9 and STAT1 were increased markedly in the late stage of FJzz1 infection and the promotion of the phosphorylation and nuclear translocation of STAT1, implicating the activation of the JAK-STAT signaling pathway during FJzz1 infection. In addition, abundant type I/III IFNs were produced after FJzz1 infection. However, type I/III IFNs and ISGs decreased greatly in FJzz1-infected LLC-PK1 cells following the silencing of the RIG-I-like receptors (RLRs), including RIG-I and MDA5, and the Toll-like receptors (TLRs) adaptors, MyD88 and TRIF. Altogether, FJzz1 infection induces the production of type-I/III IFNs in LLC-PK1 cells, in which RLRs and TLRs signaling pathways are involved, followed by the activation of the JAK-STAT signaling cascade, triggering the production of numerous ISGs to exert antiviral effects of innate immunity.

## Introduction

Porcine epidemic diarrhea (PED) is an acute and highly contagious enteric viral disease of swine caused by the porcine epidemic diarrhea virus (PEDV). Neonatal piglets are most susceptible to PEDV, characterized by severe acute watery diarrhea, vomiting, dehydration, high morbidity and high mortality (1–3). Since PED was first reported in England in 1971, the outbreak has occurred frequently in many pig-producing countries (4–10). Despite the availability of CV777-derived vaccines, outbreaks continued to increase, especially the highly virulent G2 PEDV re-emerged in China in 2010, followed by the first introduction into the United States in 2013, resulting in substantial economic losses to the pork industry worldwide (11–17). PEDV is an enveloped, positive single-stranded, plus-sense RNA virus that belongs to the genus Alphacoronavirus of the family Coronaviridae in the order Nidovirales (18). PEDV consists of about 28 Kb PEDV genome including a capped 5'-untranslated region (5'-UTR), a tailed 3'-UTR, and seven open reading frames (ORF), which encodes polymerase peptides pp1a, pp1ab, S glycoprotein, accessory protein ORF3, envelope (E), membrane (M) and nucleocapsid (N) (19, 20).

Antiviral innate immunity is regarded as the first line of defense against viral infection, and typical antiviral responses in host cells are mediated by type I interferons (IFNs), such as IFN-β (21). During viral infection and replication, RNA virus genomes replication produces double-stranded (ds) RNA, which can be recognized as a pathogen-associated molecular pattern by host pattern recognition receptors including RIG-I-like receptors (RLRs) in the cytoplasm or Toll-like receptors (TLRs) in endosomes (22, 23), leading to the synthesis and secretion of type I IFNs. Subsequently, the secretion of IFNs induces hundreds of IFN-stimulated genes (ISGs) through the Janus kinase (JAK)-signal transducer and activator of transcription (STAT) signaling pathway to act the antiviral effects (24, 25). In recent years type III IFNs have been identified as novel viral factors that target the downstream signaling pathways needed to amplify ISGs to establish a host antiviral state (26–28). Although type III IFNs bind to a receptor complex distinct from the type I IFNs receptor, type I and III IFNs have been reported to share a common downstream signaling pathway *via* JAK-STAT, leading to the induction of ISGs (29, 30).

Virus interactions with host innate immune responses drive mutual evolutionary changes, which result in the remarkable diversification of viruses and host antiviral responses (31). In the competition between virus and host cells, many viruses including coronavirus have evolved various strategies to evade or disrupt the antiviral immunity such as the type I and III IFNs (32, 33). Several viral proteins have been identified as IFN- I/III antagonists in members of the family *Coronaviridae*, including Middle East respiratory syndrome coronavirus (34, 35), mouse hepatitis virus (36, 37), severe acute respiratory syndrome coronavirus (SARS-CoV) (38) and SARS-CoV-2 (39–41), a novel emerging β-coronavirus that causes the coronavirus disease 2019 (COVID-19). PEDV belongs to α-coronavirus together with transmissible gastroenteritis virus (TGEV). Accumulating evidence showed that several PEDV proteins such as nsp1, nsp3, nsp5, nsp15, nsp16, and N could antagonize type I and/or type III productions through diverse molecular mechanisms (30, 42–45). Additionally, limited reports showed that PEDV could suppress IFNs production (43, 44, 46), but the detailed mechanism regarding IFNs inhibition remained elusive. More interestingly, some studies reported that ISGs were upregulated significantly during PEDV infection (47, 48), which were consistent with our previous results based on proteomics analysis. Therefore, further investigations are needed to elucidate the modulations of ISGs and IFNs responses in PEDV-infected cells. In the present study, we demonstrated that PEDV-infection induced IFN-I/III productions through RLRs and TLRs-mediated pathways to produce a large number of ISGs by activating the JAK-STAT signaling pathway to exert antiviral effects. This study provides new insight into understanding the modulation of host natural immune responses to PEDV infection.

## Materials and methods

### Cells and antibodies

Porcine kidney epithelial cells (LLC-PK1) used in this study were cultured in modified Eagle's medium (MEM, Life Technologies, 11095098) with 10% fetal bovine serum (FBS, Gibco, 10,099,141) at 37°C in a humidified atmosphere of 5% CO_2_. Swine testis epithelial cells (ST) and African green monkey kidney epithelial cells (MARC-145) were grown in Dulbecco' Modified Eagle's Medium nutrient (DMEM, Sigma-Aldrich, D6429) with 10% FBS at 37°C with 5% CO_2_. Anti-STAT1 antibody (14994) and anti-Phospho-STAT1 antibody (9167) were purchased from Cell Signaling Technology (CST). Anti-ISG15 antibody (ab285367) was purchased from Abcam. Anti-β-actin antibody (60,008–1), Horseradish peroxidase (HRP)-conjugated anti-mouse IgG antibody (SA00001-1), and HRP-conjugated anti-rabbit IgG antibody (SA00001-2) were obtained from Proteintech Group. The monoclonal antibody (Mab) against PEDV N protein was made in our laboratory (49). 4' 6-diamidino-2-phenylindole (DAPI, C1002) was purchased from Beyotime. poly(I:C) LMW (low molecular weight) was obtained from *In vivo*Gen.

### PEDV propagations and infections

PEDV G2 strain FJzz1 (GenBank accession no. MK288006) was previously isolated in Vero E6 cells in our laboratory (50, 51), and virus titers were determined by 50% tissue culture infective doses (TCID_50_). LLC-PK1 cells grown to approximately 90% confluence in 6-well plates were mock-infected with FBS-free MEM containing 10 μg/mL trypsin or infected with PEDV at the indicated multiplicity of infection (MOI). After incubation for 1 hour (h) at 37°C, LLC-PK1 cells were washed with phosphate-buffered saline (PBS) 3 times to remove the unattached viruses and maintained in MEM supplemented with 5 μg/ml trypsin at 37°C. ST and MARC-145 cells were mocked infected with FBS-free DMEM containing 10 μg/ml trypsin or infected with PEDV at an MOI of 0.01. After incubation for 1 h at 37°C, ST and MARC-145 cells were washed by PBS 3 times and maintained in DMEM supplemented with 10 μg/ml trypsin at 37°C. Then the cells were collected at the indicated time points for further analyses.

### Quantitative Real-time PCR

According to the manufacturer's instructions, total RNA was extracted from PEDV-infected LLC-PK1, ST and MARC-145 cells using the RNeasy Mini kit (QIAGEN, 74104) For reverse transcription (RT)-qPCR analysis, one microgram of total RNA was transcribed to cDNA using the Revert Aid First Stranded cDNA Synthesis Kit (Thermo Fisher Scientific, K1622). The synthesized cDNA was then used as the template for quantitative PCR using SYBR Green PCR mix according to the manufacturer's instructions with a LightCycler system (Roche, Switzerland). The RT-qPCR-specific primers are listed in **Table 1**. The glyceraldehyde-3-phosphate dehydrogenase (GAPDH) gene was used for each experiment's internal control. Relative transcription levels of target genes were presented as fold changes relative to the respective controls using the 2-^ΔΔCt^ threshold method.

**Table 1:** Primer sequences in this study.

Gene name	Forward primer (5’-3’)	Reverse primer (5’-3’)
Porcine-RSAD2	ATCTGCCCACCACCCCCACTA	ACGGCTCTCCGCCTGAAAAGT
Porcine-OAS1	GAGTTTTCCACCTGCTTCACG	AAATCTGTTTTCCCGCTTCCT
Porcine-Mx1	GGAGGCGGAAGAAGAAAAGAA	CAGAGGGATGTGGCTGGAGAT
Porcine-Mx2	AGAGGCAGCGGAATCATCAC	CTCCACTTTGCGGTAGCTGA
Porcine-IFIT1	AAGAAGATGAAGGAGAAAAGT	CAGGAGAGAAGAGAAGGGTGT
Porcine-ISG15	AATGTGCTTCAGGATGGGGTG	CCAGGATGCTCAGTGGGTCTC
Porcine-IRF9	ATCCTCCAGGACCCCTTCAA	AACCCTACCTTCCGGAGACT
Porcine-STAT1	TCCGTTTTCATGACCTTCTGT	CTGAATATTCCCTGACTGAGT
Porcine-IFN-β	GCTAACAAGTGCATCCTCCAAA	CCAGGAGCTTCTGACATGCCA
Monkey-IFN-β	CTAGCACTGGCTGGAATGAGACT	GGCCTTCAGGTAATGCAGAATC
Monkey-GAPDH	CCTTCCGTGTCCCTACTGCCAAC	GACGCCTGCTTCACCACCTTCT
Porcine-IFN-λ1	GGTGCTGGCGACTGTGATG	GATTGGAACTGGCCCATGTG
Porcine-IFN-λ3	ACTTGGCCCAGTTCAAGTCT	CATCCTTGGCCCTCTTGA
Porcine-IFN-λ4	GCTATGGGACTGTGGGTCTT	AGGGAGCGGTAGTGAGAGAG
Porcine-RIG-I	GTGTGCGGTGTTTCAGATGC	AGCCTGCTGCTCGGATATTT
Porcine-MDA5	CACTTGCCCGCGAATTAACA	GTCCGAGACGTCCAGACTTG
Porcine-MAVS	CCTCTGGGACCTCTTCGACA	GCTGTTTGAATTCCGCAGCA
Porcine-MyD88	CCATTCGAGATGACCCCCTG	TAGCAATGGACCAGACGCAG
Porcine-TRIF	GCTCCCGAGCTGGAGTTATC	GGTACCTGGAAATCCTCGCA
Porcine-GAPDH	ATGGTGAAGGTCGGAGTGAAC	CGTGGGTGGAATCATACTGG

### Western blotting analysis

LLC-PK1 cells infected with PEDV or mock-infected at the indicated time points were harvested and lysed on ice in RIPA Lysis and Extraction Buffer (Thermo Fisher Scientific, 89,901) supplemented with Protease Inhibitor Cocktail (Bimake, B14001) and Phosphatase Inhibitor Cocktail (Bimake, B15001) for 30 min. The cell lysates were then centrifuged at 4°C at 10,000 rpm for 10 min to remove insoluble components. Equal amounts of protein were resolved by 10% SDS-PAGE and electrophoretically transferred onto 0.2-μm nitrocellulose Western blotting membranes (GE Healthcare, 10,600,001). After blocking with 5% nonfat dry milk in tris-buffered saline-Tween (TBST) for 2 h at room temperature, the membranes were incubated with the anti-STAT1 antibody, anti-Phospho-STAT1 antibody, anti-ISG15 antibody, anti-N antibody or anti-β-actin antibody at 4°C overnight. After washing with TBST for 30 min, the membranes were incubated with the HRP-conjugated anti-rabbit IgG antibodies or HRP-conjugated anti-mouse IgG antibodies for 1 h at room temperature, followed by washing with TBST for 30 min. Signals were detected with chemiluminescence (Thermo Fisher Scientific, 34,580).

### Immunofluorescence assay

LLC-PK1 cells seeded in 6-well plates were mock-infected or infected with PEDV at an MOI of 0.01 for 18 h, as described above. Then cells were fixed with 4% paraformaldehyde for 10 min at room temperature and permeabilized using 0.1% Triton X-100 for 10 min at 4°C. After three washes with PBS, the cells were sealed with 3% bovine serum albumin (BSA) in PBS for 1 h and then incubated with anti-STAT1 antibody and anti-N antibody, followed by secondary AF488-conjugated goat anti-rabbit IgG antibody and AF594-conjugated goat anti-mouse IgG antibody in the dark for 1 h. The cell nuclei were stained with 0.1% DAPI in the dark for 15 min at room temperature. Immunofluorescence images were captured with inverted fluorescence microscopy in the dark.

### SiRNA-mediated interference

Small interfering RNA (siRNA) against pig RIG-I, MDA5, MAVS, MyD88, TRIF, and negative control (NC) siRNA referred to in the previous report were synthesized by GenePharma (Shanghai, China) (52). LLC-PK1 cells were seeded in 12-well plates, and transient transfections of siRNAs were performed using Lipofectamine™ RNAiMAX transfection reagent (13,778,075; Thermo Fisher Scientific) according to the manufacturer’s instructions. The interfering efficiency of siRNA was analyzed by RT-qPCR due to the lack of specific antibodies against pig RIG-I, MDA5, MAVS, MyD88 or TRIF. LLC-PK1 cells were transfected with specific siRNAs or NC siRNA, followed by PEDV infection, and the transcriptions of I/III IFNs and ISGs were analyzed by RT-qPCR. The siRNA sequences are listed in **Table 2**.

**Table 2:** Short interfering RNA target sequences.

Gene name	The target sequence (5’-3’)
RIG-I	CUCUUGGAGGCUUAUUUCA
MDA5	GAGAAACCAGUGAUUCCCU
MAVS	CUGCUGCGGAAUUCAAACA
MyD88	CGGCUGAAGUUAUGUGUGU
TRIF	CCACCUUUCAGAAGAAGAU

### Statistical analysis

GraphPad Prism 6 (GraphPad, La Jolla, CA, USA) was used for statistical analyses. Statistical significance was assessed with the Student' *t*-test, and data are presented as mean ± SD for at least two independent experiments. Differences were considered statistically significant (Asterisks) when the *P*-value was less than 0.05.

## Results

### Proliferation characteristics of FJzz1 in LLC-PK1 cells

**Figure 1:**
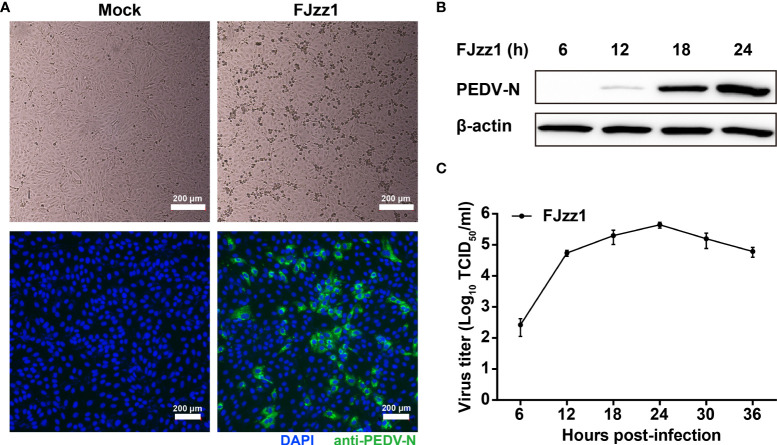
Efficient replication of FJzz1 in LLC-PK1 cells. **(A)** CPE and immunofluorescence identification of PEDV replication in LLC-PK1 cells. LLC-PK1 cells were infected with FJzz1 at an MOI of 0.01, and a CPE was observed at 18 hpi. Then cells were fixed with 4% paraformaldehyde and stained with mouse anti-N MAb. Scale bar = 200 μm. **(B)** Expression of the N protein in FJzz1-infected LLC-PK1 cells was detected by Western blotting. LLC-PK1 cells were infected with PEDV at an MOI of 0.01, and Western blotting was conducted using anti-N MAb at 6, 12, 18, and 24 hpi, respectively. **(C)** Multi-step growth kinetics of FJzz1 in LLC-PK1 cells. LLC-PK1 cells were infected with FJzz1 at an MOI of 0.01. The cell lysates were collected at the designated times and titrated with a TCID_50_ infectivity assay.

Vero cells are widely used as suitable cell models for PEDV isolation and propagation *in vitro*. However, Vero cells lost the capacity to produce type I IFNs due to a chromosomal deletion. Thus, Vero cells are considered less suitable for studies of PEDV-cell interactions, especially innate immunity, during PEDV infection. PEDV mainly infects the intestinal tract of piglets, where villous epithelial cells are regarded as the primary target cells *in vivo* for PEDV. Although IPEC-J2 is a line of porcine intestinal epithelial cells, its susceptibility to PEDV is controversial. LLC-PK1, as a kind of porcine kidney epithelial cell, is reported to be permissive to PEDV infection (30, 53, 54). To determine the proliferative kinetics of PEDV in LLC-PK1 cells, cytopathic effect (CPE), immunofluorescence assay (IFA) identification, viral protein expression, and multi-step growth curve were performed. As shown in **Figure 1A**, LLC-PK1 cells inoculated with FJzz1 developed visible CPEs at 18 h post-infection (hpi), while no CPEs were found in the mock-infected cells. In addition, LLC-PK1 cells infected with FJzz1 displayed specific green fluorescence when treated with a Mab directed against PEDV N protein, but no green fluorescence was observed in the mock-infected cells. Western blotting analysis further confirmed the productive and efficient infection of LLC-PK1 cells by FJzz1 (**Figure 1B**). Moreover, a multi-step growth curve of FJzz1 based on the TCID_50_ at different hpi was visualized. It showed that the titers of FJzz1 increased steadily during infection and reached a peak at 24 hpi (**Figure 1C**). Our results showed that FJzz1 could infect LLC-PK1 cells and have good proliferation, demonstrating that LLC-PK1 is an alternative cell line for studying innate immunity modulated by PEDV infection *in vitro*.

### FJzz1 infection induces the production of ISGs in LLC-PK1 cells

**Figure 2:**
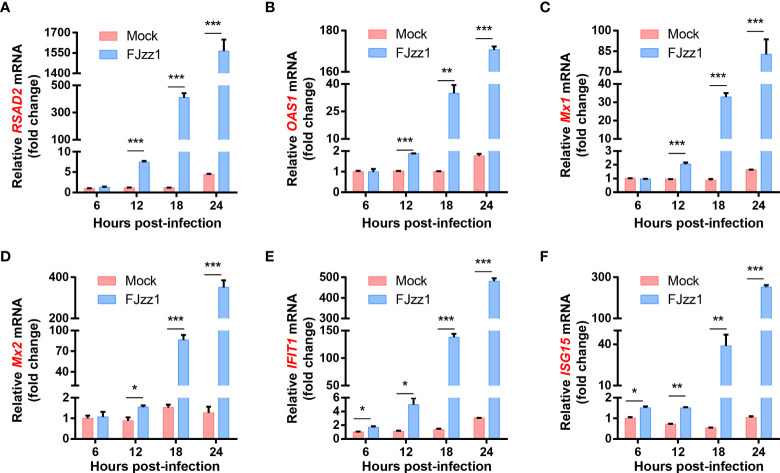
FJzz1 infection induced the production of ISGs in LLC-PK1 cells. LLC-PK1 cells were seeded in 12-well plates, followed by mock-infection or FJzz1 infection at an MOI of 0.01. Total cellular RNA was prepared at indicated times post-PEDV infection, and the mRNA levels of RSAD2 **(A)**, OAS1 **(B)** Mx1 **(C)**, Mx2 **(D)**, IFIT1 **(E)**, and ISG15 **(F)** were determined by RT-qPCR and normalized to that of porcine GAPDH. These data are representative of the results of at least two independent experiments and error bars represent standard deviations. Asterisks indicate statistical significance.*, *P* < 0.05; **, *P* < 0.01; ***, *P* < 0.001.

The interactions between PEDV and host cells have been studied previously in our laboratory using TMT relative quantitative proteomics method. The results showed that compared with mock-infected LLC-PK1 cells, ISGs including radical S-adenosylmethionine domain containing 2 (RSAD2; also known as viperin), 2',5'-Oligoadenylate synthetase 1(OAS1), the GTPase Mx proteins (Mx1 and Mx2), interferon-induced protein with tetratricopeptide repeats 1 (IFIT1), and ISG15 were upregulated significantly in FJzz1 infected LLC-PK1 cells (**Supplementary Figure 1**). To confirm the relative quantitative proteomics results, the transcriptional levels of ISGs were examined by RT-qPCR. As shown in **Figure 2**, after infection with FJzz1 at MOI of 0.01, the mRNA of ISGs climbed at 12 hpi and continued to upregulated for hundred times at 18 hpi and 24 hpi. By contrast, the mRNA of ISGs in mock-infected LLC-PK1 cells remained relatively stable at different time points. Furthermore, the mRNA expressions of ISGs induced by FJzz1 infection were upregulated in dose-dependent manners (**Supplementary Figure 2**). Together, these results suggest that FJzz1 infection induces the production of ISGs, and we speculate that FJzz1 infection may activate the IFNs signaling cascade.

### JAK-STAT signaling pathway is activated during FJzz1 infection

**Figure 3:**
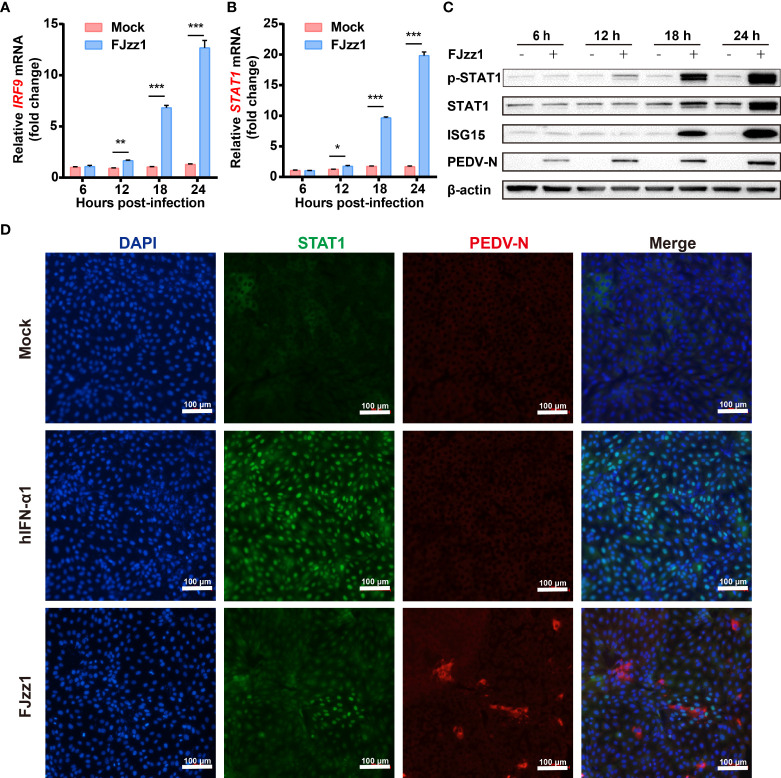
JAK-STAT signaling pathway was activated during FJzz1 infection. **(A, B)** Transcriptional levels of IRF9 and STAT1 in PEDV-infected cells. LLC-PK1 cells were infected with FJzz1 at an MOI of 0.01, and total cellular RNA was prepared at the indicated times post-infection to determine the IRF9 **(A)** and STAT1 **(B)** mRNA levels by RT-qPCR. **(C)** Expression of the phosphorylated and non-phosphorylated forms of STAT1 and the downstream ISG15 in FJzz1-infected LLC-PK1 cells. LLC-PK1 cells were infected with FJzz1 at an MOI of 0.01, and Western blotting was conducted at the indicated times post-infection using an anti-STAT1 antibody, anti-Phospho-STAT1 antibody, and anti-ISG15 antibody, respectively. **(D)** FJzz1 infection-induced STAT1 nuclear translocation. LLC-PK1 cells were infected with FJzz1 at an MOI of 0.01 for 18 h. Cells were then fixed and stained with anti-STAT1 and anti-N antibodies for 1 h. Alexa Fluor 488-conjugated goat anti-mouse secondary antibody and Alexa Fluor 594-conjugated goat anti-rabbit antibody was used to visualize STAT1, and N. Nuclei were stained with DAPI. Scale bar = 100 μm. *, P < 0.05; **, P < 0.01; ***, P < 0.001.

In general, IFNs recognition of IFN receptors on the surface of host cells activates the JAK-STAT signaling pathway to induce the production of numerous ISGs, which elicit antiviral and immune-regulatory activities. To investigate the effect of PEDV infection on the JAK-STAT signaling pathway, total RNA of FJzz1-infected LLC-PK1 cells was extracted at different time points, and RT-qPCR was conducted to explore the transcriptional levels of IRF9 and STAT1, the key molecules in the JAK-STAT signaling pathway. The results showed that the transcriptional levels of IRF9 and STAT1 were upregulated significantly at 12-24 hpi in time-dependent manners (**Figures 3A**, **B**). Western blotting analysis showed that compared with the mock-infected cells, both phosphorylated and non-phosphorylated forms of STAT1 as well as the expression of ISG15, were upregulated markedly in PEDV-infected cells, especially in the late stage of infection (18-24 hpi) (**Figure 3C**). STAT1 mediates the production of ISGs only when its nuclear translocation occurs from the cytoplasm to the nucleus. To understand the regulation of PEDV to the JAK-STAT1 signaling pathway, we then examined whether PEDV infection induced the STAT1 nuclear translocation. LLC-PK1 cells were mock-infected or infected with FJzz1, and another group of cells was treated with hIFN-α as a positive control, followed by co-staining with anti-STAT1 mAb and anti-PEDV N mAb. As shown in **Figure 3D**, STAT1 was mainly observed in the cytoplasm of mock-infected cells without hIFN-α treatment. After hIFN-α stimulation, STAT1 was mainly observed in the nuclei of mock-infected cells as anticipated. It is noteworthy that STAT1 could also be observed in the nuclei of FJzz1-infected cells, implicating the induction of STAT1 nuclear translocation by FJzz1 infection. Altogether, the JAK-STAT signaling pathway is activated during FJzz1 infection.

### FJzz1 infection induces the production of type I/III IFNs

**Figure 4:**
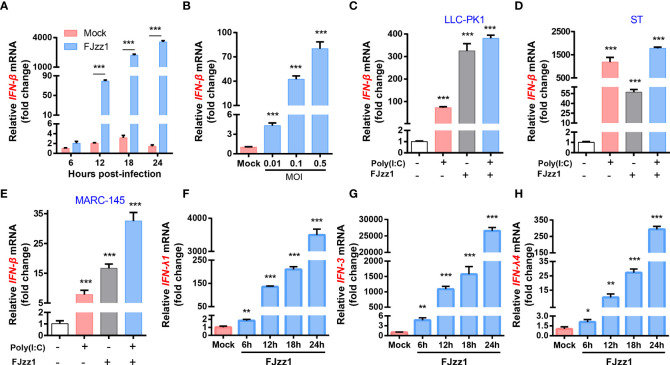
FJzz1 infection induced the production of type I and type III IFNs. **(A)** Transcriptional levels of IFN-β in PEDV-infected cells. LLC-PK1 cells were infected with FJzz1 at an MOI of 0.01, and total cellular RNA was prepared at the indicated times post-infection to determine the IFN-β mRNA level by RT-qPCR. **(B)** PEDV infection induced IFN-β production in a dose-dependent manner. LLC-PK1 cells were infected with different MOI (0.01, 0.1, 0.5) of FJzz1 for 18 h, and total mRNA was extracted to detect the IFN-β mRNA level by RT-qPCR. **(C–E)** PEDV infection induced IFN-β production in different cell lines. LLC-PK1 cells **(C)**, ST cells **(E)**, and MARC-145 cells **(E)** were infected with FJzz1 at an MOI of 0.01 for 12 h, followed by stimulation with poly(I:C) for 12 h, and total cellular RNA was extracted to detect the IFN-β mRNA level by RT-qPCR. **(F–H)** PEDV infection induced type III IFNs production. LLC-PK1 cells were infected with FJzz1 at an MOI of 0.01, and total cellular RNA was prepared at the indicated times post-infection to determine the mRNA level of IFN-λ1 **(F)**, IFN-λ3 **(G)**, and IFN-λ4 **(H)** by RT-qPCR. These data are representative of the results of at least two independent experiments and error bars represent standard deviations. Asterisks indicate statistical significance.*, *P* < 0.05; **, *P* < 0.01; ***, *P* < 0.001.

To further explore the regulation of PEDV infection on the production of type I IFN, total RNA of FJzz1-infected LLC-PK1 cells was extracted at 6, 12, 18 and 24 hpi, and RT-qPCR was performed to examine the IFN-β transcription. As shown in **Figure 4A**, after infection with FJzz1 at MOI of 0.01, the mRNA of IFN-β climbed at 12 hpi and culminated at 24 hpi, indicating time-dependent induction of IFN-β. Meanwhile, the up-regulation of FJzz1 infection on the production of IFN-β was dose-dependent (**Figure 4B**), demonstrating that FJzz1 infection induces the IFN-β production, which was contrary to previous reports (43, 44, 46). To further determine the induction of IFN-β by PEDV infection, IFN-β responses were evaluated in FJzz1-infected cells including LLC-PK1, ST and Marc-145 simultaneously. The results showed that FJzz1 could induce IFN-β production in various cell lines. Meanwhile, FJzz1 infection could also enhance the poly(I:C) induced IFN-β production (**Figures 4C**–**E**), which was in line with our conclusion that FJzz1 infection induces the IFN-β expression. Type III IFNs play a crucial role in innate antiviral immunity, especially in mucosal immunity induced by intestinal viruses (55–57). Therefore, we also examined the type III IFNs responses in FJzz1-infected LLC-PK1 cells. Interestingly, the mRNA of type III IFNs including IFN-λ1, IFN-λ3 and IFN-λ4 in FJzz1-infected LLC-PK1 cells were upregulated significantly in time-dependent manners with those in mock-infected LLC-PK1 cells (**Figures 4F**–**H**). Altogether, FJzz1 infection induces the production of type I/III IFNs in LLC-PK1 cells.

### Both RLRs and TLRs signaling pathways mediated in the production of type I/III IFNs during FJzz1 infection

**Figure 5:**
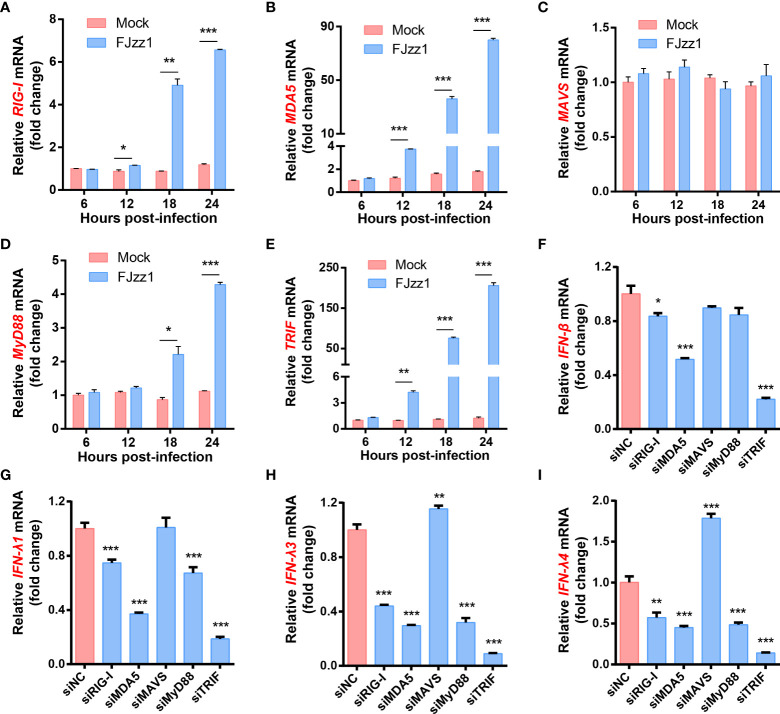
FJzz1 infection induced the production of type I/III IFNs through RLRs and TLRs signaling pathways. **(A–E)** FJzz1 infection increased the production of RIG-I, MDA5, MyD88, and TRIF. LLC-PK1 cells were infected with FJzz1 at an MOI of 0.01, and total cellular RNA was prepared at the indicated times post-infection to determine the mRNA level of RIG-I **(A)**, MDA5 **(B)**, MAVS **(C)**, MyD88 **(D)**, and TRIF **(E)** by RT-qPCR. **(F–I)** RLRs and TLRs signaling pathways mediated in the production of type I/III IFNs. LLC-PK1 cells were transfected with 80 nM specific siRNA targeting RIG-I, MDA5, MAVS, MyD88, TRIF, or an NC siRNA for 24 h, and then cells were mocked or PEDV-infected (MOI=0.01). At 18 hpi, total cellular RNA was extracted to determine the mRNA level of IFN-β **(F)**, IFN-λ1 **(G)**, IFN-λ3 **(H)**, and IFN-λ4 **(I)** by RT-qPCR. These data are representative of the results of at least two independent experiments and error bars represent standard deviations. Asterisks indicate statistical significance.*, *P* < 0.05; **, *P* < 0.01; ***, *P* < 0.001.

Antiviral responses in mammals are mediated by type I/III IFNs, which trigger hundreds of ISGs production through the JAK-STAT signaling pathway to establish the antiviral state. Upon virus infection, TLRs and RLRs signaling pathways may be activated. To determine whether TLRs and/or RLRs signaling pathways mediated in the production of I/III IFNs, transcriptional levels of the five key molecules including RIG-I, MDA5, MAVS, MyD88 and TRIF were detected. As shown in **Figures 5A**–**E**, transcriptional levels of RIG-I, MDA5, MyD88 and TRIF in FJzz1-infected LLC-PK1 cells were upregulated significantly at 12-24 hpi in a time-dependent manner, while the mRNA of MAVS did not change remarkably compared with that of the mock-infected LLC-PK1 cells. We speculated that both RIG-I/MDA5-mediated RLRs signaling pathway and MyD88/TRIF-mediated TLRs signaling pathway might be triggered by FJzz1 infection to produce type I/III IFNs. Specific siRNAs targeting RIG-I, MDA5, MAVS, MyD88, and TRIF, adaptor molecules in the RLRs and TLRs signaling pathways, were synthesized to verify this hypothesis. Transient transfection and RT-qPCR assays were conducted to assess the knockdown efficiency of each siRNA (**Supplementary Figure 3**). LLC-PK1 cells were transfected with each siRNA followed by PEDV infection to examine the transcriptional levels of type I/III IFNs under RLRs/TLRs signaling pathways interruption. The results showed that siRNAs targeting RIG-I, MDA5, and TRIF but not MAVS or MyD88 significantly reduced the mRNA of IFN-β (**Figure 5F**), demonstrating that RIG-I/MDA5-mediated RLRs signaling pathway and TRIF-mediated TLRs signaling pathway involved in the production of IFN-β during FJzz1 infection. Moreover, the transcriptional levels of IFN-λ including IFN-λ1, IFN-λ3 and IFN-λ4 were diminished markedly in cells transfected with siRIG-I, siMDA5, siMyD88 or siTRIF but not MAVS, compared with the siNC following FJzz1 infection (**Figures 5G**–**I**), demonstrating that RIG-I/MDA5-mediated RLRs signaling pathway and MyD88/TRIF-mediated TLRs signaling pathway involved in the production of type III IFNs during FJzz1 infection. Altogether, both RLRs and TLRs signaling pathways mediated in the production of type I/III IFNs during FJzz1 infection.

### The expression of ISGs induced by FJzz1 infection depends on the production of type I/III IFNs

**Figure 6:**
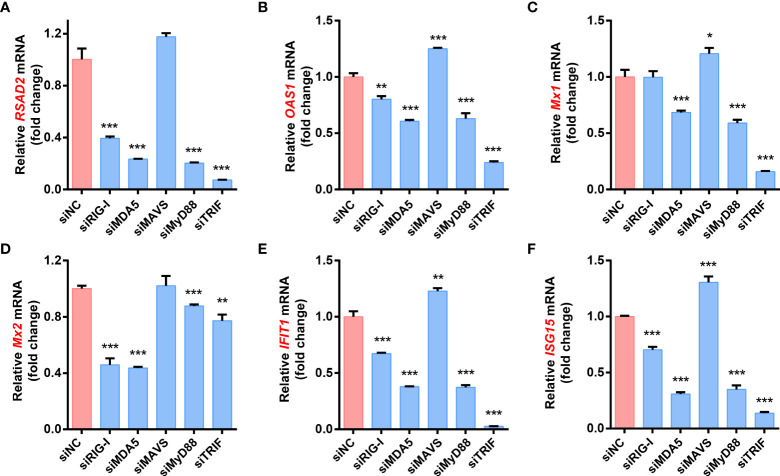
RLRs and TLRs signaling pathways are involved in the production of ISGs during FJzz1 infection. LLC-PK1 cells were infected with FJzz1 at an MOI of 0.01 for 18 h, and total cellular RNA was extracted to determine the mRNA level of RSAD2 **(A)**, OAS1 **(B)**, Mx1 **(C)**, Mx2 **(D)**, IFIT1 **(E)**, and ISG15 **(F)** by RT-qPCR. These data are representative of the results of three independent experiments and error bars represent standard deviations. Asterisks indicate statistical significance.*, *P* < 0.05; **, *P* < 0.01; ***, *P* < 0.001.

To further determine the effect of RLRs/TLRs signaling on PEDV-induced ISGs production, LLC-PK1 cells were transfected with specific siRNAs targeting adaptor molecules in the RLRs and TLRs signaling pathways such as RIG-I, MDA5, MAVS, MyD88 and TRIF, followed by FJzz1 infection, and the expression of various ISGs was analyzed by RT-qPCR. The results showed that knockdown of RIG-I, MDA5, MyD88, or TRIF significantly decreased FJzz1-induced ISGs mRNA expression, including RSAD2, OAS1, Mx1, Mx2, IFIT1, and ISG15 (**Figures 6A**–**F**). Interestingly, we noticed that the transcriptional levels of these ISGs did not decrease or even increase in cells transfected with siRNA targeting MAVS compared with cells transfected with siNC, which was consistent with the appreciable trends of the production of type I/III IFNs. These results further demonstrate that the RIG-I/MDA5-mediated RLRs signaling pathway and MyD88/TRIF-mediated TLRs signaling pathway are involved in the production of ISGs during FJzz1 infection. Considering the consistent production trends between ISGs and type I/III IFNs, we think the expression of ISGs induced by FJzz1 infection depends on the production of type I/III IFNs.

## Discussion

The first line of defense against viral infection and replication is the innate immune system, in which ISGs are the major components for the establishment of a host antiviral state (58). Many ISGs have been reported to suppress viral replication through diverse mechanisms (59–63). In the present study, we found that the mRNA expressions of ISGs including RASD2, OAS1, Mx1, Mx2, IFIT1, and ISG15 in FJzz1-infected LLC-PK1 cells were upregulated significantly in dose-dependent manners, indicating that the innate immune responses mediated by ISGs were induced and activated by FJzz1 infection. Remarkably, RSAD2 and ISG15 were confirmed to inhibit CV777 replication in Vero E6 cells through functional analysis (64). Therefore, we speculated that the abundant ISGs induced by PEDV infection might display excellent antiviral activities. Although the invasion of PEDV could not induce efficient ISGs transcription in the early stage of infection (6 hpi), the mRNA of ISGs increased for hundred times in the late stage of FJzz1 infection (18-24 hpi) (**Figure 2**). Coincidently, the up-regulation induced by PEDV infection could also be found in other studies. For example, the mRNA expression of the Mx1 gene in PEDV-infected IPEC-J2 cells was significantly increased after 48 h of CV777 infection as measured by RT-qPCR, and the transcription trend of the Mx1 gene was parallel with the increase of viral RNA (65). In addition, several ISGs were identified to increase significantly in porcine jejunum tissues in response to a virulent strain of PEDV and its attenuated strain through comparative proteome analysis (47). Moreover, both endoribonuclease-deficient PEDV and wild-type PEDV infections induce robust expression of ISGs and proinflammatory cytokines in PK1 cells (30). However, PEDV proliferation was too fast that the adequate ISGs produced by host cells failed to inhibit PEDV replication in the late stage of infection. The interplay between PEDV infection and the host’s innate immune responses is so complicated that further investigations are needed to elucidate the modulation of ISGs during PEDV infection.

The production of ISGs mainly depends on the activation of the JAK-STAT signaling pathway, in which the recruited upstream signals activate JAK1 and Tyk2, leading to the phosphorylation of the STAT proteins, such as STAT1 and STAT2. Subsequently, phosphorylated STAT1 and STAT2 form heterodimers and then combine with IRF9 to form IFN-stimulated gene factor 3 (ISGF-3), a transcriptional complex that could translocate into the nucleus and bind to the IFN-stimulated response elements, resulting in the production of abundant ISGs (66, 67). In this study, we observed that the transcriptional level of STAT1 was increased in FJzz1-infected LLC-PK1 cells, and the consistent conclusion could also be reported in PEDV-infected Vero and IPEC-J2 cells in other studies (65, 68). Moreover, compared with the mock-infected group, both phosphorylated and non-phosphorylated forms of STAT1 were upregulated significantly in FJzz1-infected LLC-PK1 cells in time-depended manners. Conversely, Guo et al. demonstrated that STAT1 expression was markedly reduced in a proteasome-dependent manner without inhibiting STAT1 transcription in PEDV-infected Vero and IPEC-J2 cells (68). In our perspective, these conflicting results are most likely due to the different cell lines or the different PEDV strains. With these in mind, more integrated and detailed studies of strain-resolved or even single-cell-resolved responses to the JAK-STAT signaling pathway are needed to aid in understanding the modulations of host innate immune response to PEDV infection.

In terms of different cell lines involved in the interaction between host and PEDV infection, Vero cells are widely used as a suitable cell model for PEDV isolation and propagation *in vitro* (8, 69). However, they are not suitable for studies of PEDV-cell interactions, especially innate immunity during PEDV infection, because of the chromosomal defectiveness of type I IFNs (70, 71). Zhang et al. demonstrated that MARC-145, a subline of African green monkey kidney cells, was an alternative cell line that supports PEDV replication with typical CPE characterized by syncytium formation (43), which was similar to that in Vero cells. PEDV mainly infects the intestinal tract of piglets, where villous epithelial cells are regarded as the primary target cells *in vivo* for PEDV. IPEC-J2, a nonhomogeneous porcine intestinal epithelial cell line, was isolated from the jejunum of neonatal piglets. Although several studies reported the susceptibility of IPEC-J2 cells to PEDV (46, 68, 72), we found that IPEC-J2 cells possessed very low efficiencies of infection by PEDV through IFA and Western blotting experiments. Coincidentally, the controversial susceptibility of IPEC-J2 cells to PEDV was also reported in other studies (44, 73). Considering the species specificity and efficiency of infection, we determined to use LLC-PK1, a kind of porcine kidney epithelial cells, to study the innate immune responses upon PEDV infection. In this study, our results showed that FJzz1 could infect LLC-PK1 cells and have good proliferation, which was in line with other studies (30, 53, 54). Moreover, LLC-PK1 cells have been identified to have the ability to express a high level of ISGs and type I/III IFNs in this and other studies (30, 53), demonstrating that LLC-PK1 cells are a suitable cell model for the study of the interactions between innate immunity and PEDV infection *in vitro*. Recently, the nonhomogeneous IPEC-J2 cells were subcloned by limited serial dilutions to obtain a homogeneous cell population, named IPEC-DQ, which were susceptible to PEDV infection with high efficiency and expressed high levels of ISGs and type I/III IFNs (44, 45, 74). Therefore, IPEC-DQ cells may be the optimal cell model for the study of innate immunity possibly modulated by enteric coronavirus including PEDV and TGEV in the future.

To establish persistent infection, viruses have developed diverse molecular mechanisms to evade or disrupt the host’s innate immune response, particularly by inhibition or disruption of IFN signaling cascades (38, 75, 76). PEDV, as a member of the Coronaviridae, also encodes some proteins that serve as IFNs antagonists. PEDV papain-like protease 2 was reported firstly as a type I IFN antagonist, which negatively regulated RIG-I and STING mediated IFN-β expression (77). PEDV N protein antagonizes IFN-β production by sequestering the interaction between IRF3 and TBK1 (42), and PEDV-encoded 3C-like protease, nsp5 regulates its IFN antagonism by cleaving NEMO (78). It is worth noting that nsp1 can inhibit type I and III IFNs production by different molecular mechanisms (43, 44), indicating the important role of nsp1 in suppressing innate immunity upon PEDV infection. Moreover, PEDV nsp15 can directly degrade the RNA levels of TBK1 and IRF3 dependent on its endoribonuclease activity to suppress IFN production and constrain the ISGs, by which PEDV antagonizes the host innate response to facilitate its replication (79). Recently, a study reported that PEDV nsp7 antagonized IFN-α-induced IFN signaling by competing with KPNA1 for binding to STAT1, thereby revealing a new mechanism developed by PEDV to inhibit the type I IFN signaling pathway (80) These identified IFN antagonists may contribute to the delay of type I/III IFNs transcriptions in the early stage of PEDV infection. However, the present study showed that the mRNA of type I/III IFNs was upregulated significantly in a time-depend manner, suggesting that these antagonists could not limit the production of type I/III IFNs, especially in the late stage of infection. Further studies demonstrated that RLRs and TLRs-mediated pathways were involved in the high levels of type-I/III IFNs productions, which triggered abundant ISGs by activating the JAK-STAT signaling pathway. It is confusing that the adequate type-I/III IFNs and ISGs transcription failed to suppress PEDV replication in the late stage, although many type-I/III IFNs antagonists were highly expressed at the same time. Interestingly, this surprising phenomenon could also be found in TGEV infection (25). In this study, we proposed for the first time that FJzz1 did not suppress type-I/III IFNs induction at the early stage of infection and induced abundant type-I/III IFNs production in the peak of FJzz1 replication, which was contrary to several reports that PEDV infection inhibited type-I/III IFNs production (43, 44, 46). In our opinion, these differences may result from the different PEDV strains. So far, most of the strains used to study the interaction between PEDV and host innate immune responses have been concentrated in CV777, AJ1102, USA/Colorado/2013, and PC22A strains, which had 97.1%, 99.1%, 98.9%, and 98.9% nucleotide homology with FJzz1, respectively (**Supplementary Table 1**). Moreover, compared with the identified type I/III IFNs antagonists including nsp3, nsp15, and nsp16 of CV777 (77, 79, 81), nsp5, nsp7, and N of AJ1102 (42, 78, 80), nsp1, nsp14 and nsp15 of USA/Colorado/2013 (30, 43–45), as well as nsp16 of PC22A (82), many amino acid mutations occurred in those of FJzz1. We speculate that these mutations between FJzz1 and other PEDV strains may lead to the difference in modulations of host innate immune responses to PEDV infection, which should be confirmed by further experiments, including reverse genetic analyses. Meanwhile, we observed the induction of type-I/III IFNs in LLC-PK1 cells infected by FJzz1-F200 (**Supplementary Figure 3**), an attenuated FJzz1 strain that was obtained *via* serially passaging *in vitro* (51), suggesting that the attenuated FJzz1-F200 may have the potential for developing PEDV live-attenuated vaccines.

In summary, our data demonstrate for the first time that FJzz1 infection induces the production of type-I/III IFNs in LLC-PK1 cells, which depends on the mediation of RLRs and TLRs signaling pathways. Subsequently, the downstream JAK-STAT signaling cascade is activated, triggering the production of a large number of ISGs to exert antiviral effects. This study provides new insight into the interaction between PEDV and host’s natural immune responses.

## Data availability statement

The original contributions presented in the study are included in the article/**Supplementary Material**. Further inquiries can be directed to the corresponding authors.

## Author contributions

PC and YZ conceived and designed the experiments. PC, JZ, and JY performed the experiments. PC, RL, and ML analyzed the data. LY, FG, YJ, CL, and WT contributed reagents/materials/analysis tools. HL, GT, and YZ participated in analysis and discussion. PC wrote the paper. YZ and GT checked and finalized the manuscript. All authors contributed to the article and approved the submitted version.

## Funding

The study was supported by the National Natural Science Foundation of China (32102657, 31472207), the Shanghai Youth Scientific and Technological Yang Fan Program Grant (20YF1457800), the China Postdoctoral Science Foundation (2020M670555), Shanghai Municipal Agriculture Science and Technology Project (2020-02-08-00-08-F01465), and China Agriculture Research System of MOF and MARA (NYCYTX-009).

## Conflict of interest

The authors declare that the research was conducted in the absence of any commercial or financial relationships that could be construed as a potential conflict of interest.

## Publisher’s note

All claims expressed in this article are solely those of the authors and do not necessarily represent those of their affiliated organizations, or those of the publisher, the editors and the reviewers. Any product that may be evaluated in this article, or claim that may be made by its manufacturer, is not guaranteed or endorsed by the publisher.

## Supplementary material

Supplementary Figure 1 The comparison of the dysregulated proteins in FJzz1-infected LLC-PK1 cells. LLC-PK1 cells were mock-infected or infected with FJzz1 at an MOI of 0.01 for 18 h, and all cells were collected to perform the TMT labeled comparative proteomic analysis. The heatmap shows the relative expression level of the dysregulated proteins in both Mock- and FJzz1-infected LLC-PK1 cells (Log_2_ fold-change).

Supplementary Figure 2 FJzz1 infection induced the production of ISGs in LLC-PK1 cells in a dose-dependent manner. LLC-PK1 cells were infected with different MOI (0.01, 0.1, 0.5) of FJzz1 for 18 h, and total cellular RNA was extracted to determine the mRNA level of RSAD2 **(A)**, OAS1 **(B)**, Mx1 **(C)**, Mx2 **(D)**, IFIT1 **(E)**, and ISG15 **(F)** by RT-qPCR. These data are representative of the results of three independent experiments, and error bars represent standard deviations. Asterisks indicate statistical significance. ***, *P* < 0.001.

Supplementary Figure 3 SiRNA interference. LLC-PK1 cells were transfected with 80 nM specific siRNA targeting RIG-I, MDA5, MAVS, MyD88, TRIF, or an NC siRNA for 24 h, and then cellular RNA was extracted for analysis of RIG-I **(A)**, MDA5 **(B)**, MAVS **(C)**, MyD88 **(D)**, and TRIF **(E)** mRNA levels by RT-qPCR. These data are representative of the results of three independent experiments, and error bars represent standard deviations. Asterisks indicate statistical significance. ***, *P* < 0.001.

Supplementary Figure 4 The attenuated strain FJzz1-F200 infection induced the production of type I and type III IFNs. **(A–D)** Transcriptional levels of IFN-β, IFN-λ1, IFN-λ3, and IFN-λ4 in PEDV-infected cells. LLC-PK1 cells were infected with FJzz1-F200 at an MOI of 0.01, and total cellular RNA was prepared at 18 hpi to determine the mRNA level of IFN-β **(A)**, IFN-λ1 **(B)**, IFN-λ3 **(C)** and IFN-λ4 **(D)** by RT-qPCR. These data are representative of the results of three independent experiments and error bars represent standard deviations. Asterisks indicate statistical significance. ***, *P* < 0.001.

Supplementary Table 1 Nucleotide and amino acid homology analysis of the FJzz1 strain.
