# Supplementary figures and images for: Porcine epidemic diarrhea virus strain FJzz1 infection induces type I/III IFNs production through RLRs and TLRs-mediated signaling
Source: Front Immunol. 2022 Jul 25;13:984448. doi: 10.3389/fimmu.2022.984448 (PMC9357978; doi:10.3389/fimmu.2022.984448)

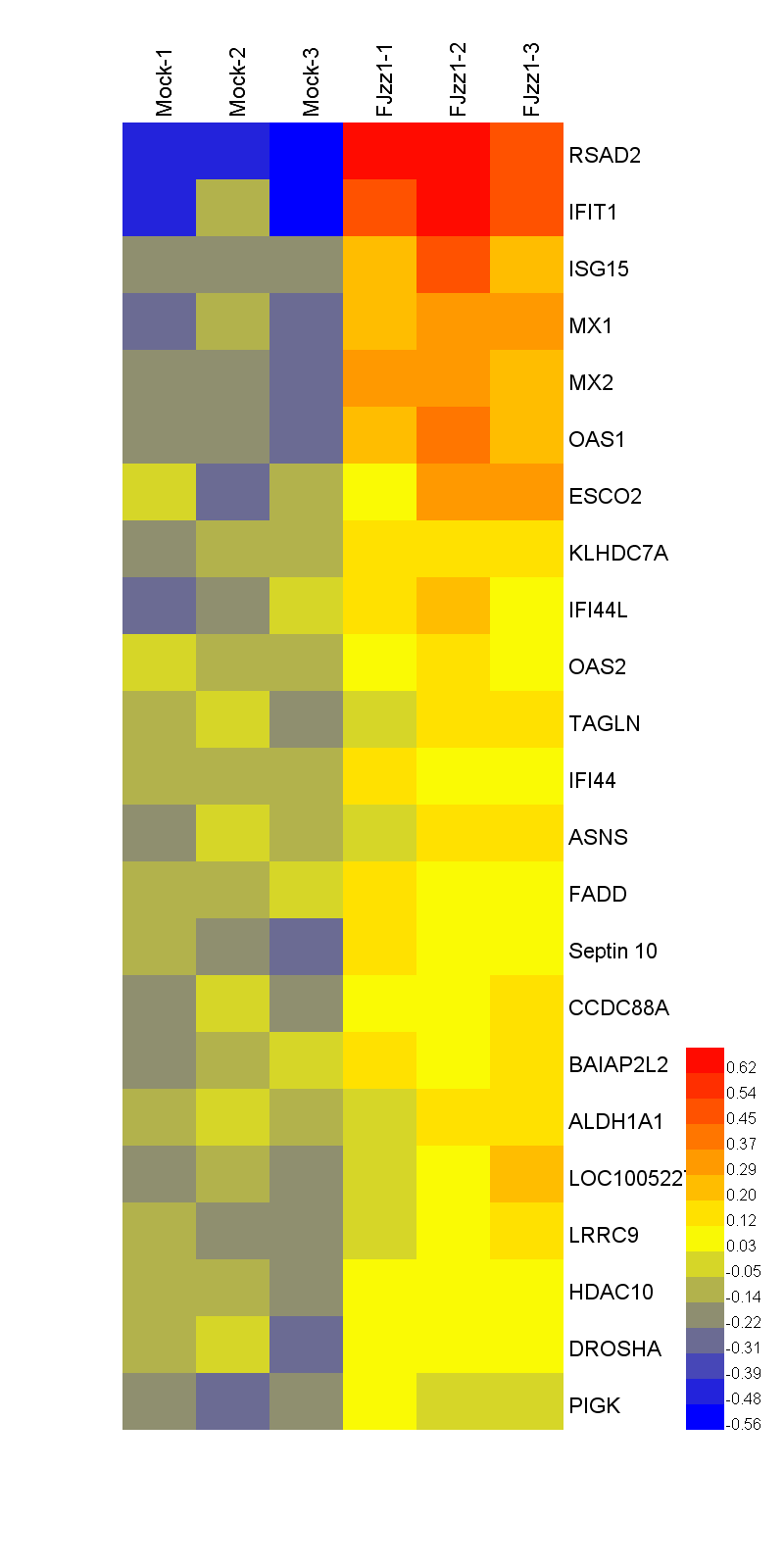

Supplement: Supplementary Figure 1 — The comparison of the dysregulated proteins in FJzz1-infected LLC-PK1 cells. LLC-PK1 cells were mock-infected or infected with FJzz1 at an MOI of 0.01 for 18 h, and all cells were collected to perform the TMT labeled comparative proteomic analysis. The heatmap shows the relative expression level of the dysregulated proteins in both Mock- and FJzz1-infected LLC-PK1 cells (Log2 fold-change). [file Image_1.tiff]

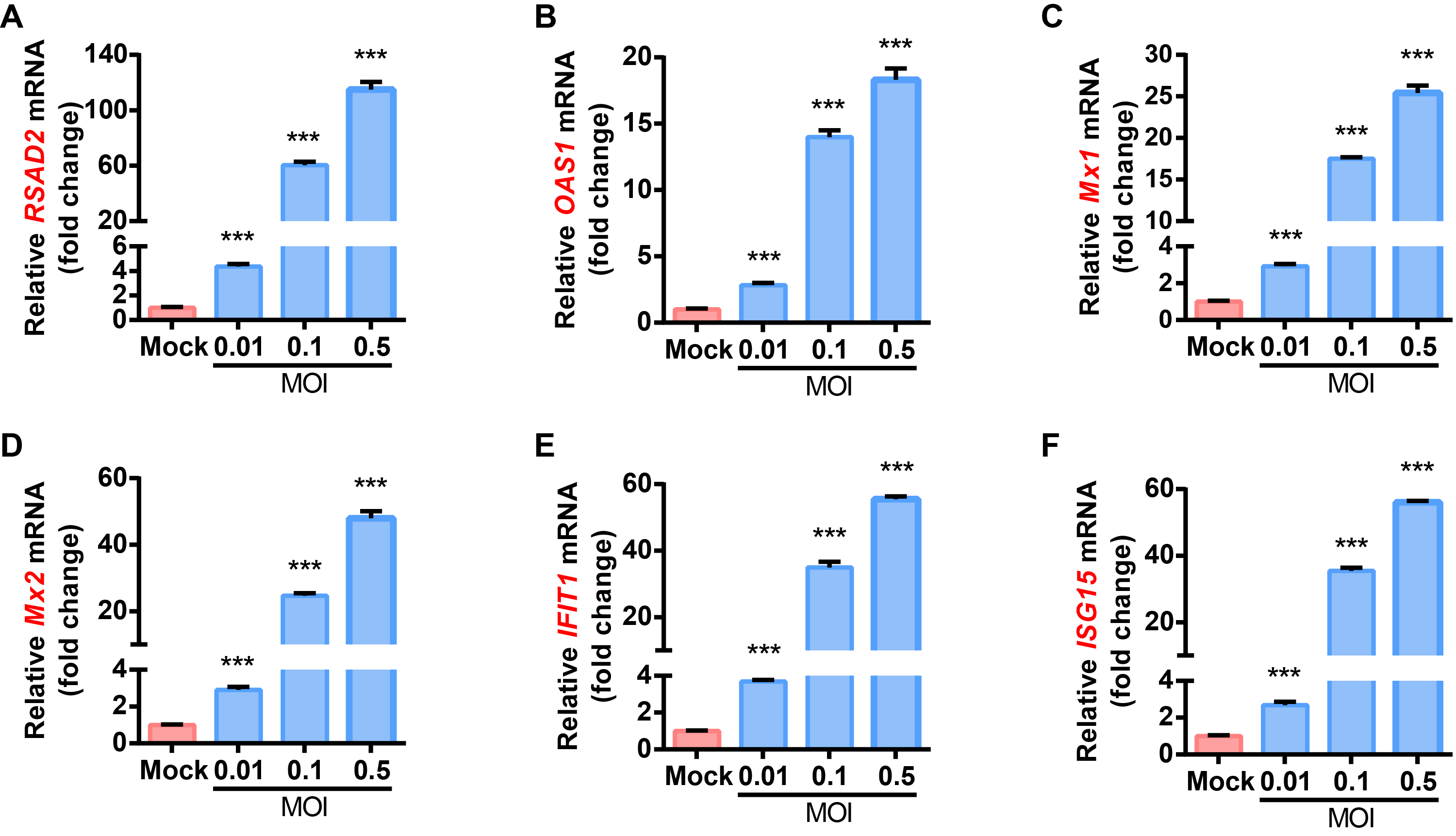

Supplement: Supplementary Figure 2 — FJzz1 infection induced the production of ISGs in LLC-PK1 cells in a dose-dependent manner. LLC-PK1 cells were infected with different MOI (0.01, 0.1, 0.5) of FJzz1 for 18 h, and total cellular RNA was extracted to determine the mRNA level of RSAD2 (A), OAS1 (B), Mx1 (C), Mx2 (D), IFIT1 (E), and ISG15 (F) by RT-qPCR. These data are representative of the results of three independent experiments, and error bars represent standard deviations. Asterisks indicate statistical significance. ***, P < 0.001. [file Image_2.tif]

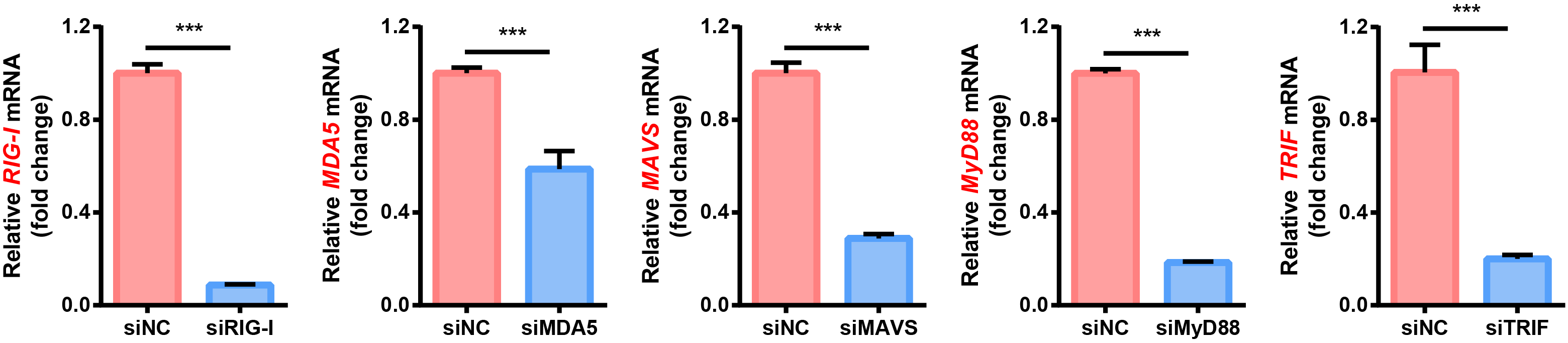

Supplement: Supplementary Figure 3 — SiRNA interference. LLC-PK1 cells were transfected with 80 nM specific siRNA targeting RIG-I, MDA5, MAVS, MyD88, TRIF, or an NC siRNA for 24 h, and then cellular RNA was extracted for analysis of RIG-I (A), MDA5 (B), MAVS (C), MyD88 (D), and TRIF (E) mRNA levels by RT-qPCR. These data are representative of the results of three independent experiments, and error bars represent standard deviations. Asterisks indicate statistical significance. ***, P < 0.001. [file Image_3.tif]

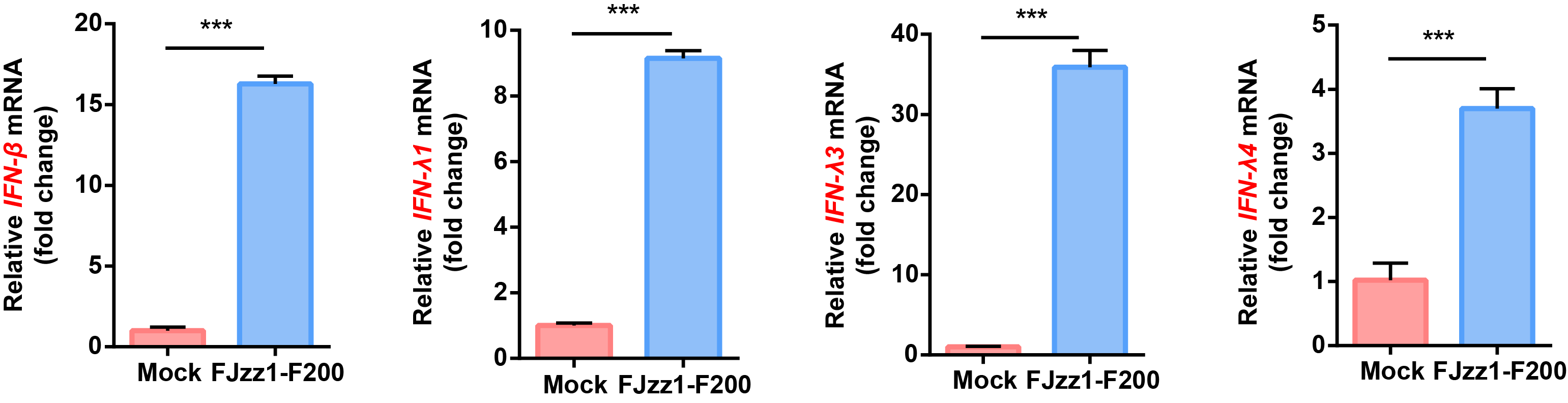

Supplement: Supplementary Figure 4 — The attenuated strain FJzz1-F200 infection induced the production of type I and type III IFNs. (A–D) Transcriptional levels of IFN-β, IFN-λ1, IFN-λ3, and IFN-λ4 in PEDV-infected cells. LLC-PK1 cells were infected with FJzz1-F200 at an MOI of 0.01, and total cellular RNA was prepared at 18 hpi to determine the mRNA level of IFN-β (A), IFN-λ1 (B), IFN-λ3 (C) and IFN-λ4 (D) by RT-qPCR. These data are representative of the results of three independent experiments and error bars represent standard deviations. Asterisks indicate statistical significance. ***, P < 0.001. [file Image_4.tif]
