# Supplementary material for: Porcine epidemic diarrhea virus strain FJzz1 infection induces type I/III IFNs production through RLRs and TLRs-mediated signaling
Source: Front Immunol. 2022 Jul 25;13:984448. doi: 10.3389/fimmu.2022.984448 (PMC9357978; doi:10.3389/fimmu.2022.984448)
Supplement: Supplementary Table 1 — Nucleotide and amino acid homology analysis of the FJzz1 strain. [file Table_1.docx]

**Supplementary Table 1. Nucleotide and amino acid homology analysis of the FJzz1 strain**

| Strains  (GenBank no.) | Nucleotide  homology | IFN-Ⅰ/Ⅲ  antagonists | Amino acid  homology | Numbers of  mutated AA |
| --- | --- | --- | --- | --- |
| CV777  (KT323979) | 97.1% | nsp3 | 97.2% | 54/1656 |
|  |  | nsp15 | 99.7% | 1/339 |
|  |  | nsp16 | 99.0% | 3/301 |
| AJ1102  (JX188454) | 99.1% | nsp5 | 99.7% | 1/302 |
|  |  | nsp7 | 100% | 0/84 |
|  |  | N | 97.3% | 12/441 |
| USA/Colorado/2013  (KF272920) | 98.9% | nsp1 | 98.2% | 2/110 |
|  |  | nsp14 | 100% | 0/517 |
|  |  | nsp15 | 99.1% | 3/339 |
| PC22A(KY499262) | 98.9% | nsp16 | 99.7% | 1/301 |
